# Attending live sporting events predicts subjective wellbeing and reduces loneliness

**DOI:** 10.3389/fpubh.2022.989706

**Published:** 2023-01-04

**Authors:** Helen Keyes, Sarah Gradidge, Nicola Gibson, Annelie Harvey, Shyanne Roeloffs, Magdalena Zawisza, Suzanna Forwood

**Affiliations:** School of Psychology and Sport Science, Anglia Ruskin University, Cambridge, United Kingdom

**Keywords:** sports spectatorship, wellbeing, loneliness, life satisfaction, worthwhile life

## Abstract

**Introduction:**

This study explored whether attending live sporting events (LSEs) improved subjective wellbeing and loneliness, above and beyond demographic predictors.

**Methods:**

Secondary data from 7,249 adults from the Taking Part 2019–20 survey (UK household survey of participation in culture and sport) were analyzed. Multiple linear regressions captured the effect of attending LSEs (yes/no) on wellbeing variables (happiness, anxiety, a sense that life is worthwhile and life satisfaction) and loneliness, with gender, Index of Multiple Deprivation (IMD), age group, health and employment as covariates.

**Results:**

For life satisfaction, a sense that life is worthwhile, and loneliness, inclusion of LSE attendance in the model improved model fit significantly, although ΔR^2^ values were small (ΔR^2^ = 0.001–0.003). For happiness and anxiety, the inclusion of LSE attendance did not alter model fit. LSE attendance was associated with increased life satisfaction (*b* = 0.171, *p* < 0.001), a greater sense of life being worthwhile (*b* = 0.230, *p* < 0.001), and reduced loneliness (*b* = −0.083, *p* < 0.01).

**Conclusion:**

LSE attendance has positive associations with some aspects of subjective wellbeing (life satisfaction and a sense of life being worthwhile) and loneliness, above and beyond demographic predictors. Whilst the variance explained is small, it is comparable to demographic predictors (e.g., being in employment). As even small-sized differences in SWB can have meaningful outcomes (e.g., for mortality), we conclude that LSE attendance may still offer a scalable, accessible and effective means of improving the public's wellbeing and reducing loneliness.

## Introduction

Loneliness has been described as a “modern behavioral epidemic” (1), exerting significant negative impacts on mental and physical health [e.g., (2–5)]. By contrast, subjective wellbeing (SWB) has been shown to be of great importance in improving mental and physical health (6–8). Within this paper, we follow the UK Office for National Statistics in defining subjective wellbeing (SWB) as involving positive affect(e.g., happiness), negative affect (e.g., anxiety), meaning and purpose in life (e.g., a sense that life is worthwhile) and life satisfaction (9).

### Passive sports engagement

Participating actively in sports offers a fruitful avenue to improve SWB and decrease loneliness that transcends demographics [e.g., (10–12)]. That is, previous literature suggests direct sports participation, known as *active* sports engagement, is linked to reduced mental distress and better mental health [e.g., (10)]. However, there is growing literature on *passive* sports engagement (e.g., attendance at live sporting events, LSEs; watching sports on TV), which has been associated with greater happiness than active sports engagement (13). Research has shown that sports spectatorship is associated with increased SWB across all ages at racket sports events (14), associated with both short-term and long-term SWB in college students (15), and associated with life satisfaction (16) and happiness (13).

Passive sports engagement may have such positive effects due to its relational nature. As social identification and self-categorization have been shown to be salient components of sport (17), it is likely that passive sports participation, by providing opportunities for regular social interaction, creates improved experiences of group identity and belonging. In support, passive sports engagement has been shown to mitigate loneliness (18) and identifying with a sports team has been found to improve social connections and thus enhance SWB (19). For example, for older adults, attending local sports team events increases emotional support, which in turn increases a sense of belonging and thus SWB (20).

### Demographic predictors

SWB and loneliness are both informed by demographic variables, and these variables plausibly predict attendance at LSEs and are therefore possible confounders in any observational analysis. Deprivation–a measure of living circumstances, income, education/skills/training, crime, barriers to housing and services, and living environment (21)–has typically been linked to lower SWB [e.g., (22, 23)] and higher loneliness (24, 25). Gender and age have also both been shown to impact SWB (20, 26, 27). Furthermore, health (28) and employment status (29) are both known predictors of SWB. These variables could plausibly impact LSE attendance, either through access (health), affordability (employment, deprivation), or motivation (age, gender), and are therefore candidate confounders that we will control for (30). In light of this literature, our analysis controls for the following demographics: gender, Index of Multiple Deprivation (IMD), age group, self-reported poor health, and being in employment.

### Current limitations

Whilst the above literature suggests LSE attendance offers potential improvement of SWB, it suffers from several gaps. Firstly, the existing research lacks generalizability by focusing narrowly on: (a) specific sports [e.g., baseball; (31); racket sports; (14)], (b) single dimensions of SWB [e.g., life satisfaction; (16); happiness; (13, 32)], and (c) subgroups in the population [e.g., college students; (15); older adults; (20)]. Secondly, this literature predominantly explores the relationship between LSE attendance and SWB, and, to the authors' best knowledge, there is little research on the association between LSE attendance and *loneliness*. Given that SWB and loneliness are negatively correlated with each other [e.g., (24, 25), LSE attendance may be theoretically linked to higher SWB *and* lower loneliness, but this is yet to be investigated. Finally, and importantly, little is known about whether passive sports engagement, such as LSE attendance (14), has beneficial effects on SWB above and beyond demographics such as deprivation and gender. Crucially, for attendance at LSEs to be a meaningful intervention, we first need to know the magnitude of the benefits of attendance at LSE in comparison with known demographic effect sizes.

As a result, our novel study is the first to address these gaps in the literature. We explore the relationship between attendance at any (vs. specific) LSEs on multiple (vs. single) dimensions of SWB (positive affect; negative affect; meaning and purpose in life; life satisfaction), as well as loneliness, within a nationally representative sample of adults (vs. specific subgroups), independent of known demographic variables. As such, our study is the first to consider LSE attendance as a possible fruitful avenue to improve SWB and decrease loneliness that transcends the more stable demographics already associated with SWB and loneliness [e.g., (10–12)].

### Study aims

Our key research question asks: Does LSE attendance predict a range of SWB measures and loneliness *above and beyond* demographic predictors, including gender, deprivation, age group, health and employment? We predict that LSE attendance will be associated with greater SWB (**H1**) and lower loneliness (**H2**), over and above demographic predictors.

## Methods

### Participants

Participants were drawn from the Taking Part Survey: A face-to-face household survey of a random representative sample of adults aged 16 or over living in England, commissioned by the Department for Digital, Culture, Media and Sport (33). We used data from Year 13 (April 2019–March 2020), which are the latest data publicly available. The data from the Taking Part survey are openly available from UK Data Service. SN: 8745, http://doi.org/10.5255/UKDA-SN-8745-1. Please note, this data was collected prior to the COVID-19 pandemic. Out of the 7,502 adult participants surveyed, 293 participants (3.9%) were missing data on covariates, so they were excluded from analyses. These exclusions left a total sample of 7,209 participants for our main analyses. See Table 1 for participant characteristics.

**Table 1:** Participant characteristics.

**Characteristic**	***N* (%) (Total *N* = 7,209)**
Female *N* (%)	3,913 (54.3%)
Age group, *N* (%)	
16–19	193 (2.7%)
20–24	300 (4.2%)
25–34	1,041 (14.4%)
35–44	1,179 (16.4%)
45–54	1,130 (15.7%)
55–64	1,191 (16.5%)
65–74	1,206 (16.7%)
75–84	736 (10.2%)
85+	233 (3.2%)
IMD decile, *N* (%)	
1 (most deprived)	740 (10.3%)
2	725 (10.1%)
3	618 (8.6%)
4	653 (9.1%)
5	774 (10.7%)
6	707 (9.8%)
7	771 (10.7%)
8	777 (10.8%)
9	781 (10.8%)
10 (least deprived)	663 (9.2%)
Poor health	
1 Very good	2,146 (29.8%)
2 Good	2,946 (40.9%)
3 Fair	1,483 (20.6%)
4 Bad	462 (6.4%)
5 Very bad	172 (2.4%)
In employment	3,971 (55.1%)
LSE attendance, yes *N* (%)	2,397 (33.3%)
Satisfaction, mean (SE)	7.76 (0.022)
Happy, mean (SE)	7.64 (0.025)
A sense that life is worthwhile, mean (SE)	8.00 (0.021)
Anxiety, mean (SE)	2.79 (0.035)
Loneliness, mean (SE)	2.22 (0.014)

### Measures

#### Demographics

Participants reported demographic details, including age group (categorical; see Table 1) and gender (male, female). Participants also supplied their home postcode, which was used to determine the level of deprivation of their home address [part of the index of multiple deprivation; IMD; (21, 34)]. The IMD (using participants' home address only) was divided into deciles, where one is the most deprived and 10 is the least deprived. Finally, participants were asked to self-report poor health “*How is your health in general?”* (Table 1 for response options, higher score indicating poorer health), and employment “*Are you working?”* (Options: “*Working”* or “*Not Working”*).

#### LSE attendance

Participants were asked a single-item question about their attendance at LSEs: “*In the last 12 months, have you attended any live sporting events?*” (Options: “*Yes*” and “*No*”).

#### SWB

Participants provided answers to four single-item SWB questions all on 0–10 Likert scales. Life satisfaction was measured by “*Overall, how satisfied are you with your life nowadays”* (0 = “*not at all satisfied?”* to 10 = “*completely satisfied”*). A sense of life being worthwhile was measured by “*To what extent do you feel that the things in your life are worthwhile?”* (0 = “*not at all worthwhile” and* 10 = “*completely*”). Happiness was measured by “*Taking all things together, how happy would you say you are?*” (0 = “*extremely unhappy*” to 10 = “*extremely happy*”). Finally, anxiety was measured by “*On a scale where 0 is “not at all anxious” and 10 is “completely anxious,” overall, how anxious did you feel yesterday?*”. These four questions were analyzed individually as single items as per ONS guidance (9).

#### Loneliness

Loneliness was measured with a single-item question “*How often do you feel lonely?*” with the response options of one “*often or always*,” two “*some of the time*,” three “*occasionally*,” four “*hardly ever*” and five “*never*.” We reverse-scored loneliness so that higher scores represent greater loneliness.

### Analysis

We used a hierarchical regression model to address the question of whether our identified demographic variables predict a range of SWB measures (satisfaction, happiness, a sense that life is worthwhile and anxiety), as well as loneliness, and whether attendance at LSEs predicts scores on these dependent measures above and beyond effects of the demographic predictors. For the demographic variables, the assumption of multicollinearity was checked using VIF: all VIF were between 1 and 1.35 and therefore acceptable [VIF>2.5 indicates high collinearity; (35)]. For each of our SWB and loneliness measures, a sequential regression method of entry [also known as hierarchical or blockwise entry; (35)] was carried out. In each analysis, IMD decile, gender (dummy coded, reference “female”), age group, poor health, and employment (dummy coded, reference “not working”) were entered in the first block, and the additional variable of attendance at LSEs (dummy coded, reference “no attendance”) was entered in the second block. These regression results are presented in Table 2. All analyses were conducted using Jamovi (1.6.23) software (36).

**Table 2:** Co-efficient (standard error) from hierarchical multiple regressions predicting satisfaction, happiness, sense of life being worthwhile, anxiety and loneliness with regression coefficients (β) specified for all predictor variables at each block of the regression.

**Predictor variables**	**Life satisfaction**	**Happiness**	**Sense of life being worthwhile**	**Anxiety**	**Loneliness**
**Block 1**					
Intercept	8.669 (0.094)^***^	8.493 (0.113)^***^	8.629 (0.095)^***^	2.446 (0.162)^***^	2.186 (0.066)^***^
Gender	−0.076 (0.040)	−0.147 (0.047)^**^	−0.238 (0.040)^***^	−0.380 (0.068)^***^	−0.207 (0.028)^***^
IMD decile	0.017 (0.007)^*^	0.013 (0.009)	0.018 (0.007)^*^	−0.020 (0.012)	−0.018 (0.005)^***^
Age group	0.113 (0.012)^***^	0.122 (0.014)^***^	0.100 (0.012)^***^	−0.237 (0.020)^***^	−0.053 (0.008)^***^
Poor health	−0.763 (0.022)^***^	−0.712 (0.026)^***^	−0.586 (0.022)^***^	0.780 (0.037)^***^	0.289 (0.015)^***^
In employment	0.080 (0.046)	0.005 (0.055)	0.167 (0.046)^***^	−0.015 (0.078)	−0.193 (0.032)^***^
*R*^2^	0.167	0.106	0.112	0.073	0.077
*F*	288^***^	171^***^	182^***^	112.8^***^	120^***^
**Block 2**					
Intercept	8.615 (0.095)^***^	8.472 (0.115)^***^	8.56 (0.096)^***^	2.458 (0.164) ^***^	2.212 (0.067) ^***^
Gender	−0.103 (0.040)^*^	−0.158 (0.048)^**^	−0.274 (0.040)^***^	−0.374 (0.069)^***^	−0.194 (0.028)^***^
IMD decile	0.014 (0.007)^*^	0.012 (0.009)	0.014 (0.004)^*^	−0.021 (0.012)	−0.017 (0.005)^***^
Age group	0.116 (0.012)^***^	0.123 (0.014)^***^	0.104 (0.012)^***^	−0.238 (0.020)^***^	−0.054 (0.008)^***^
Poor health	−0.755 (0.022)^***^	−0.709 (0.026)^***^	−0.575 (0.022)^***^	0.779 (0.037)^***^	0.285 (0.015)^***^
In employment	0.067 (0.046)	−0.0001 (0.055)	0.150 (0.046)^**^	−0.018 (0.078)	−0.186 (0.032)^***^
LSE attendance	0.171 (0.043)^***^	0.067 (0.052)	0.230 (0.044)^***^	−0.038 (0.076)	−0.083 (0.031)^**^
*R*^2^	0.168	0.106	0.116	0.073	0.078
*F*	243^***^	143^***^	157^***^	94^***^	102^***^
**Comparison**					
Δ*R*^2^	0.002	< 0.001	0.003	< 0.001	< 0.001
*F*	15.6^***^	1.63	27.5^***^	0.262	7.30^**^

^*^p < 0.05; ^**^p < 0.01, ^***^p < 0.001.

Reference categories are female, not in employment and no attendance at LSEs.

## Results

### Subjective wellbeing

#### Life satisfaction

Participants' reported life satisfaction was significantly predicted by attendance at LSEs, above and beyond the variance explained by gender, deprivation, age group, poor health and employment. Inclusion of LSE attendance in the model explained an additional 0.2% of variance in life satisfaction scores compared to the effects of gender, deprivation, age group, health, and employment, *F*_(1, 7202)_ = 15.6, *p* < 0.001. Attendance at LSEs predicted the same increase in life satisfaction (0.171) as a 1 SD increase in age group (~20 years, 0.116, see Table 2). In the final stage of the model, gender, deprivation, age group, poor health, and LSE attendance were all significant predictors of life satisfaction, with those who had attended an LSE in the last year, women, those in less deprived areas, older age groups, and those in better health reporting higher life satisfaction. Approximately 16.8% of the variance in life satisfaction was accounted for by the final model, *R*^2^ = 0.168, *F*_(6, 7202)_ = 243, *p* < 0.001. These findings are in line with **H1**.

#### Happiness

Contrary to **H1**, the addition of LSE attendance to the model did not add significantly more predictive power to the model. The final model shows that women, those in older age groups, and those in better health reported being happier, and with 10.6% of the variance in happiness scores being explained by this model, *R*^2^ = 0.106, *F*_(6, 7202)_ = 143, *p* < 0.001.

#### Sense of life being worthwhile

**Figure 1:**
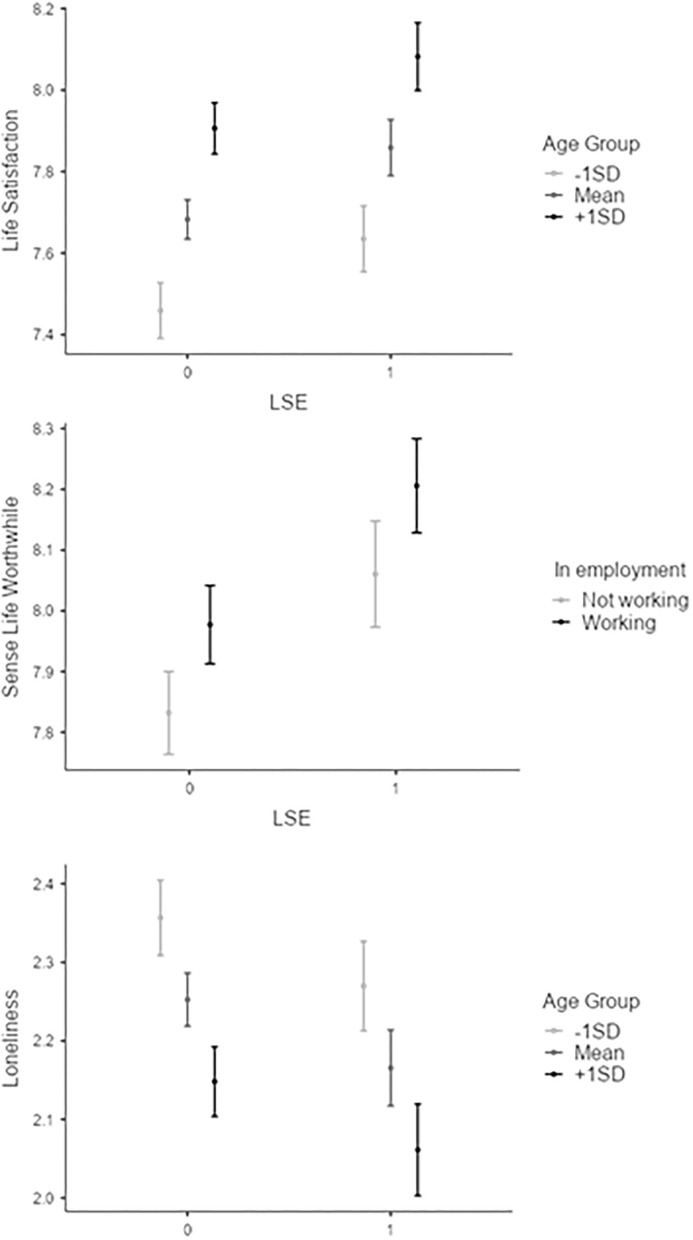
Estimated marginal means for subjective wellbeing (SWB - life satisfaction, a sense that life is worthwhile), and loneliness by age group or employment and attendance at live sporting events (LSE). These plots indicate the magnitude of the effects seen alongside demographic predictors with comparable effect sizes. Estimates are provided for age group at 1 standard deviation below the mean (−1 SD, 25–34 years), at the mean (age group 45–54 years) and at 1 standard deviation above the mean (+1 SD, 65–74 years). Error bars indicate 95% confidence intervals for estimated marginal mean estimates.

Participants' sense of whether their life was worthwhile was significantly predicted by attendance at LSEs, above and beyond the effects of gender, deprivation, age group, poor health or being in work. Indeed, the inclusion of LSE attendance in the model explained an additional 0.3% of variance in sense of life being worthwhile scores compared to the effects of gender, deprivation, age group, poor health and being in work, *F*_(1, 7202)_ = 27.5, *p* < 0.001. Attendance at LSE predicted a similar increase in a sense of a worthwhile life (0.230) as being female (0.274) and greater than that associated with being in employment (0.150, see Figure 1). In the final stage of the model, all six variables were significant predictors of a sense of life being worthwhile, with those who had attended an LSE in the last year, women, those living in less deprived areas, older age groups, those in better health, and those in employment all reporting a higher sense that life is worthwhile. The final model accounts for 11.6% of variance in sense of life being worthwhile scores, *R*^2^ = 0.116, *F*_(6, 7202)_ = 157, *p* < 0.001. Again, these findings are in line with **H1**.

#### Anxiety

Contrary to **H1**, the addition of attendance at LSEs did not provide any additional predictive power to the model. Here, men, older age groups and those with better health reported experiencing less anxiety, with health having the biggest effect with 7.3% of the variance in anxiety scores, *R*^2^ = 0.073, *F*_(6, 7202)_ = 94, *p* < 0.001 (see Table 2).

### Loneliness

Lastly, attendance at LSEs significantly predicted loneliness scores above and beyond the effects of gender, deprivation, age group, poor health or being in work. Here, the inclusion of LSE attendance in the model accounted for an additional 0.09% of the variance in loneliness scores above the variance accounted for in stage one of the model, *F*_(1, 7173)_ = 7.30, *p* = 0.007. Attendance at LSE predicted the same decrease in loneliness (−0.083) as 1 SD increase in age group (~20 years, −0.054), but less than that associated with being in employment (−0.186) or 1 SD increase in health (−0.285, see Table 2). In the final stage of the model, gender, deprivation, age group, poor health, employment and LSE attendance were all significant predictors of loneliness scores, with those who attended an LSE in the last year, men, those living in less deprived areas, older age groups, those in better health, and those in employment all reporting less loneliness. Approximately 7.8% of the variation in loneliness can be accounted for by the final model, *R*^2^ = 0.078, *F*_(6, 7173)_ = 102, *p* < 0.001. These findings are in line with **H2**.

## Discussion

The current study aimed to determine whether the benefits of attending LSEs on SWB and loneliness were impactful above and beyond demographic predictors. Supporting our predictions, LSE attendance accounted for additional variance for two aspects of SWB (life satisfaction and a sense of life being worthwhile; **H1**) and loneliness (**H2**), compared with demographic predictors alone. Specifically, LSE attendance predicted greater life satisfaction, greater sense of life being worthwhile and lower loneliness. These novel findings corroborate and extend current literature [e.g., (14, 20)], by utilizing a representative sample incorporating various age groups (age range 16–85), utilizing more than one measure of SWB and also considering loneliness and not being restricted to a specific sport.

Whilst statistically significant, the variances explained for life satisfaction, a sense that life is worthwhile and anxiety were low, indicating that real-world effects of LSE attendance on these variables may be small. However, the amount of variance explained is mostly comparable to known demographic predictors. For example, the contribution of LSE attendance to life satisfaction and loneliness was comparable to that of age. Moreover, the contribution of LSE attendance to the sense that life is worthwhile was comparable to being female and was greater than being in employment. However, the contribution of LSE attendance to loneliness was smaller than that of being in employment or having better health. Even with low variance explained, these findings may still have clinical relevance for the population. For instance, longitudinal research across the lifespan shows that higher life satisfaction predicts greater student engagement in adolescents (37), improved stress and daily affect (38), reduced life-limiting conditions and better physical health in working-age adults (39), successful aging (40) and reduced incidence of dementia in older adults (41). Additionally, studies in varied populations have shown that greater life satisfaction is associated with lower mortality rates (40, 42, 43). One such study, using the same measure of life satisfaction as in the present study, found that “one scale point higher in life satisfaction was associated with a 34.0% reduction in mortality hazards” [40, p. 10], or an increase in survival probability over the 20-year study period from 85 to 90% (43). As such, even a small increase in SWB, especially in life satisfaction as reported here, likely has significant implications for multiple profound health outcomes including mortality. Therefore, while real-world effects of LSE attendance may be small, LSE attendance could still meaningfully contribute to the public's SWB (as measured by life satisfaction and a sense that life is worthwhile) and reduced loneliness.

Contrary to **H1**, LSE attendance did not explain more variance in anxiety compared to demographic predictors alone. Despite our study measuring negative affect in identical ways to previous literature [e.g., (14, 20)], this finding contradicts prior research which has instead demonstrated significant negative relationships between sports spectatorship and negative affect (14, 15, 20). The discrepancy in findings may therefore arise from other differences between the studies, including analyzing attendance at *any* LSEs in the current study vs. only one type in previous studies [e.g., racket sports; (14)], or sampling all adults here instead of older adults only (20).

Contrary to **H1**, LSE attendance also did not explain more variance in happiness compared to demographic predictors alone. This finding may arise from happiness being retrogressive (44), whereby changes in happiness are typically short-lived and individuals rapidly return to baseline. As the current study measured LSE attendance within the last 12 months, any impact on happiness may have been short-lived and thus not evident within the dataset.

### Demographic variables

Demographic variables largely predicted SWB and loneliness in line with previous research. Specifically, being in better health [see also (28)] and within older age groups [see also (20)] both predicted greater SWB across all four measures and reduced loneliness. Additionally, those living in less deprived areas reported a greater sense that life is worthwhile, higher life satisfaction, and lowered loneliness, mostly in line with previous research [e.g., SWB, (22, 23); loneliness, (24, 25)]. Furthermore, being employed was associated with a greater sense that life is worthwhile and reduced loneliness [see also (29)]. Finally, gender predicted all dimensions of SWB and loneliness. Specifically, women reported greater life satisfaction, happiness, and a sense of life being worthwhile, but also greater anxiety and loneliness than men. Thus, gender had differential relationships with SWB dimensions, which may explain mixed literature on the relationship between gender and SWB [e.g., (20, 45)], whereby the relationship between gender and SWB depends upon the exact type of SWB.

### Strengths, limitations and directions for future research

This paper utilizes a large, nationally representative sample of UK participants, which strengthens generalizability of the findings. However, due to the survey's cross-sectional design, we cannot establish causality. Future research should therefore determine causality through a longitudinal or experimental approach. Promisingly, recent research suggests sports participation has a four times bigger causal effect on life satisfaction than life satisfaction has on sports participation (46).

Given the exploratory nature of this study, other relevant factors associated with LSE attendance were not included. For instance, Funk et al. (47) specify that the type of sporting event and motivation for attendance both account for variability in SWB. Indeed, Stieger et al. (48) found that SWB increased among football spectators only when they supported the team they were spectating. Additionally, LSEs within the current study could vary from a school sports day (non-prestige events) to a premiership league football game (prestige events). As non-prestige activities have fewer barriers to entry [i.e., not involving funds or transport; (49)], such activities may be particularly attractive to unemployed individuals or those from deprived backgrounds (whom we found had low SWB). Therefore, understanding underlying motives for LSE attendance, whilst also considering accessibility of sporting events, could aid identification of specific sporting events that are (a) more strongly correlated with increased SWB and reduced loneliness and (b) accessible to more vulnerable groups.

### Implications

Our findings have practical implications by indicating that people across demographics may derive some wellbeing benefits from attending LSEs (e.g., increased life satisfaction, a greater sense that life is worthwhile and reduced loneliness). Although small, LSE attendance explains similar variance to known demographic predictors, and even small changes in SWB can have meaningful real-world impacts (e.g., on mortality). Thus, passive sports engagement may be a fruitful avenue for future impactful interventions. Current initiatives [e.g., the “Sport for all of us” strategy, (50); the British government's Sporting Future strategy, (51)] largely focus on active sports engagement. The current study indicates such initiatives could also encourage passive sports engagement.

Additionally, the present study found that LSE attendance had a positive association with the sense that life was worthwhile comparable in magnitude to being employed. As such, interventions supporting and encouraging access to LSE could be especially of benefit in improving a sense that life is worthwhile for unemployed people, thereby possibly buffering against negative impacts of unemployment (29, 52). Whilst unemployment rates are dropping month-by-month in the UK and are in fact at their lowest since 1974 (53), 3.6% of the UK adult population (a significant minority of approximately 1.2 million people aged 16+) are still unemployed as of November 2022 (54). Worryingly, unemployment is known to worsen both mental and physical health (29). However, a greater sense that life is worthwhile has been linked to positive outcomes like better social engagement, improved mental health more broadly, and more engagement in behaviors to protect one's physical health (55). The current findings have implications for helping the unemployed as LSE attendance appears to improve their sense that life is worthwhile and thus may possibly help their social relationships, physical health and broader mental health (55). Further research could fruitfully test these possibilities directly. In addition, future research should explore causal effects of LSE attendance (vs. non-attendance) on a sense that life is worthwhile for unemployed (vs. employed) people, as the current findings indicate that LSE attendance may act as an effective buffer against the negative effects of unemployment. That is, by attending LSEs, unemployed people may be able to enjoy the same level of a sense that life is worthwhile as employed people who do not attend LSEs.

## Conclusion

In a large, nationally representative sample, LSE attendance has small but significant associations with greater life satisfaction and the sense that life is worthwhile, along with reduced loneliness. These predictive relationships hold above and beyond those of recognized demographic predictors and, crucially, their magnitudes are comparable to those of known demographic variables. Whilst small, these effects can have meaningful implications for the population (e.g., for mortality). As such, LSE attendance may present an accessible, scalable and effective tool for improving the public's wellbeing and reducing loneliness.

## Data availability statement

Publicly available datasets were analyzed in this study. This data can be found here: https://beta.ukdataservice.ac.uk/datacatalogue/doi/?id=8745#!#0.

## Ethics statement

Ethical review and approval was not required for the study on human participants in accordance with the local legislation and institutional requirements. The patients/participants provided their written informed consent to participate in this study.

## Author contributions

HK and SF: design, data analysis, results, and discussion. SG: design, data analysis, results, introduction, and discussion. NG, AH, SR, and MZ: design, introduction, and discussion. All authors contributed to the article and approved the submitted version.
